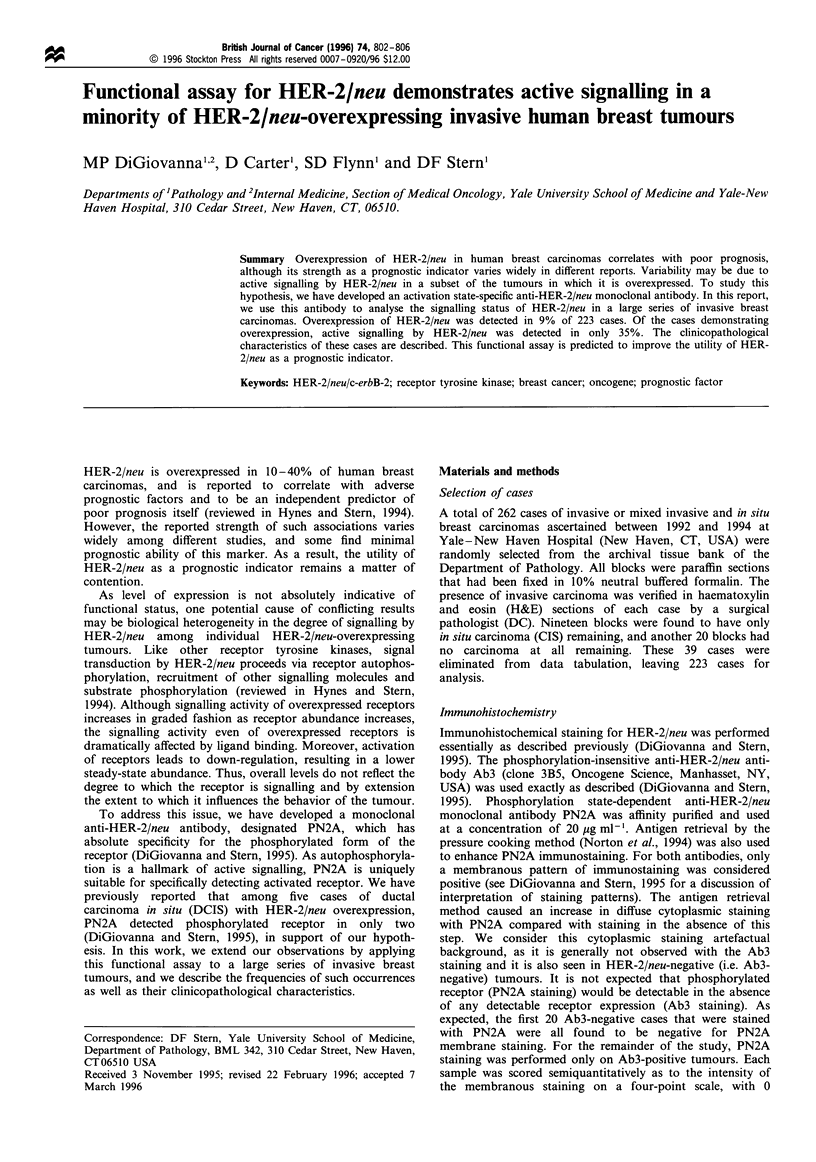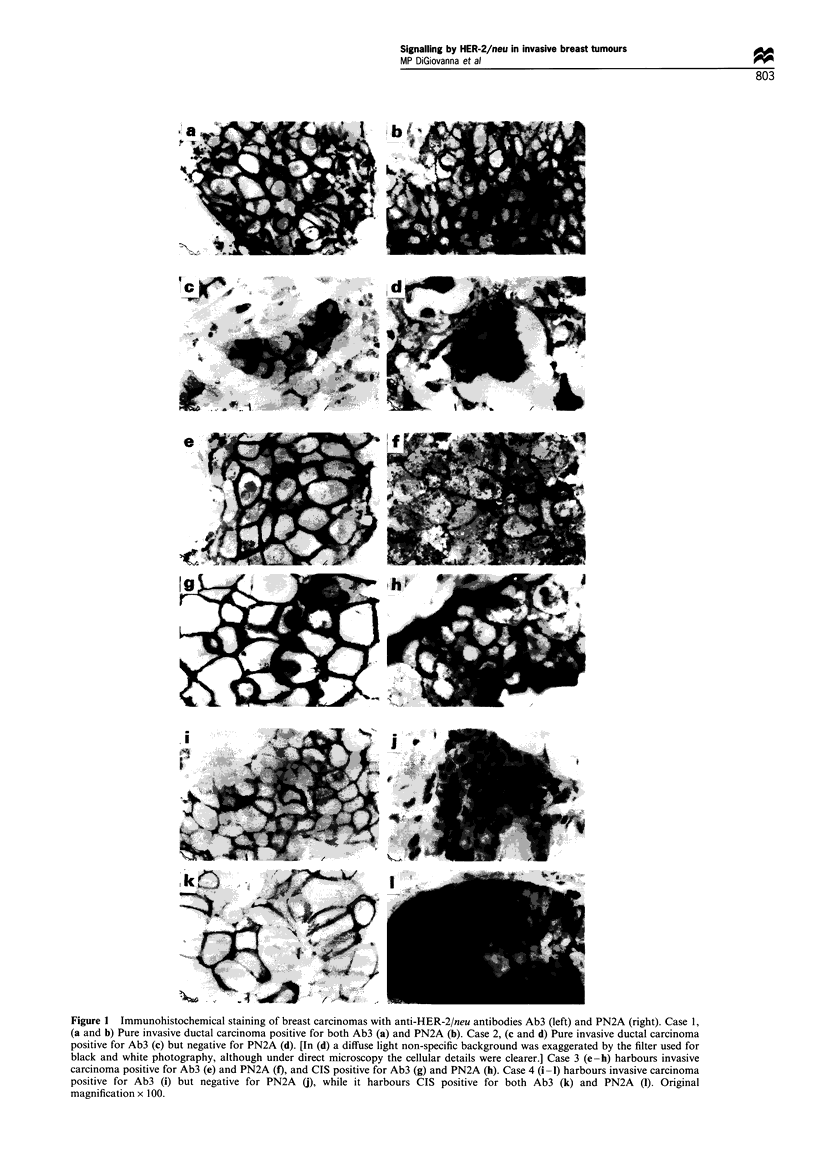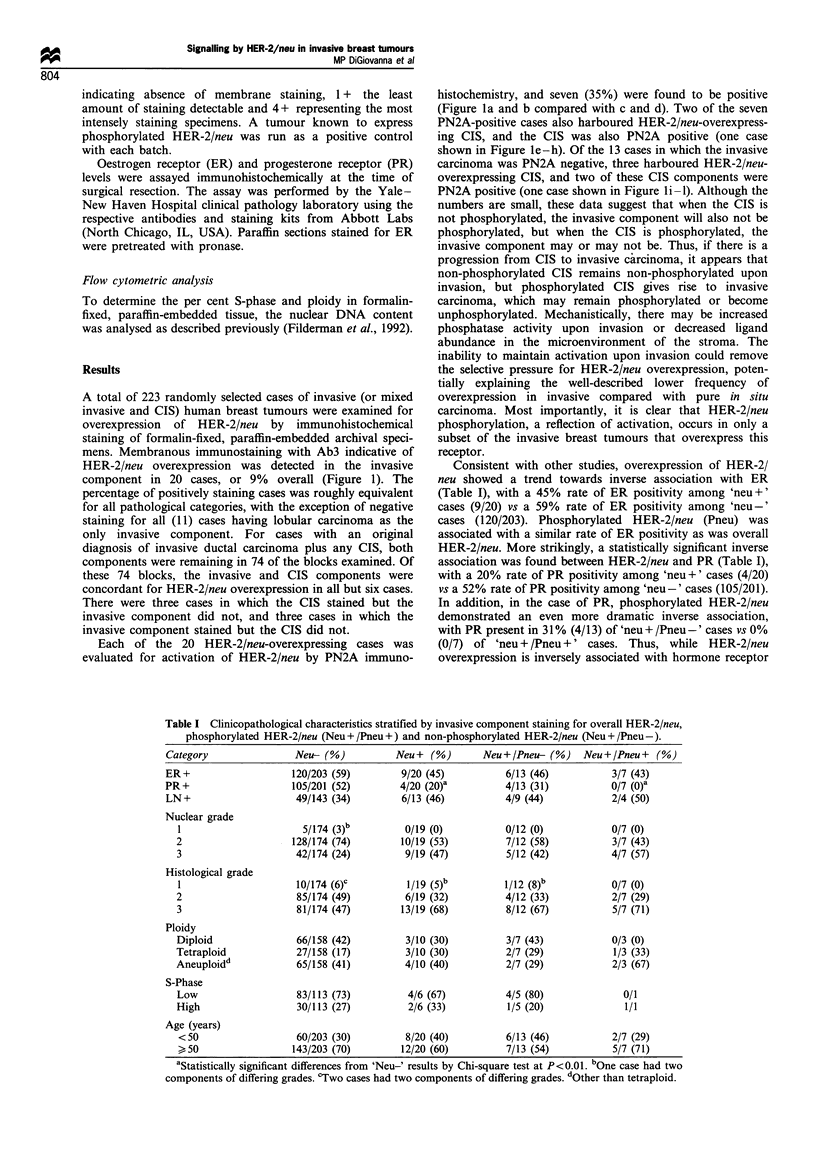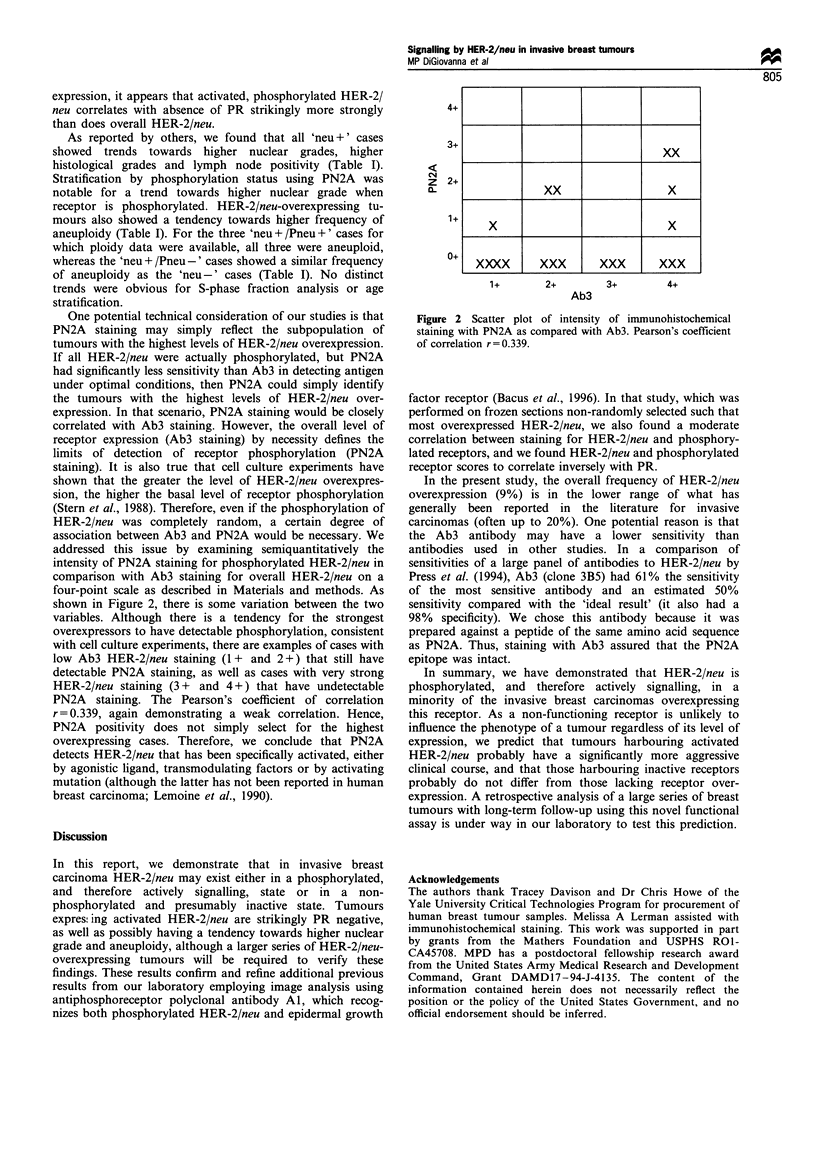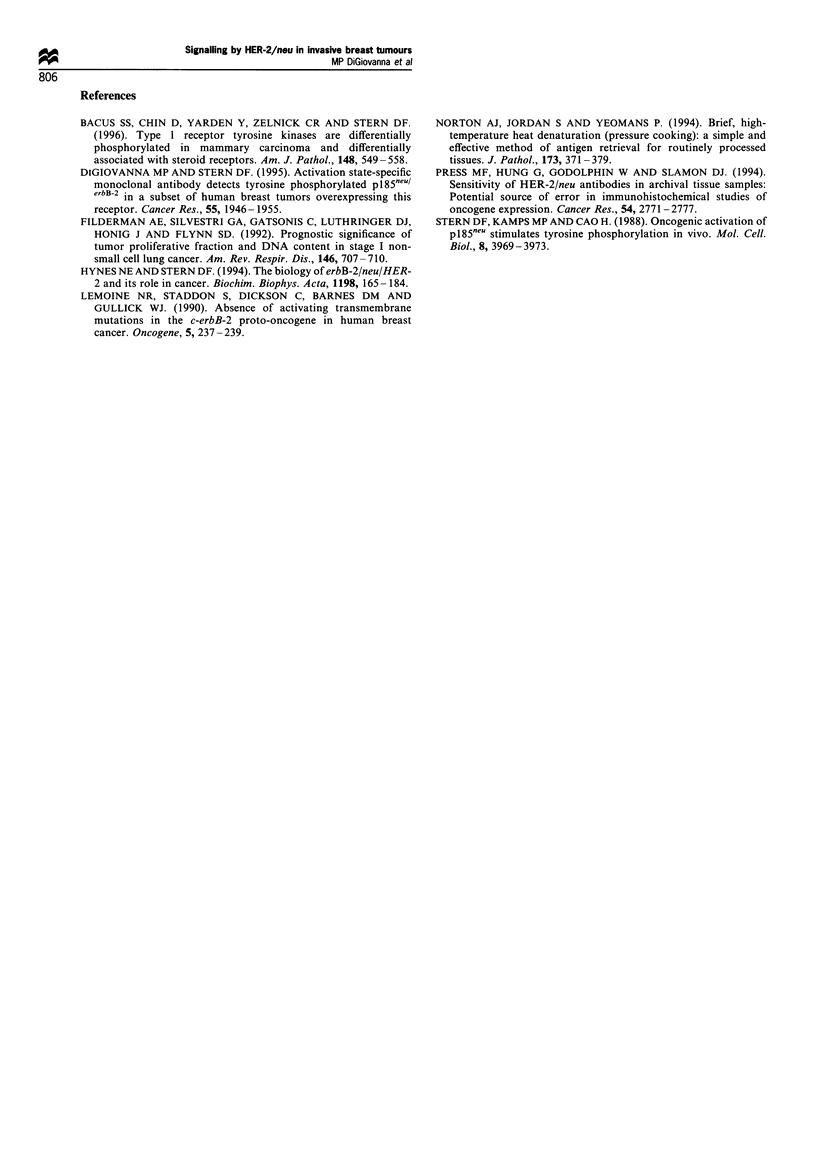# Functional assay for HER-2/neu demonstrates active signalling in a minority of HER-2/neu-overexpressing invasive human breast tumours.

**DOI:** 10.1038/bjc.1996.439

**Published:** 1996-09

**Authors:** M. P. DiGiovanna, D. Carter, S. D. Flynn, D. F. Stern

**Affiliations:** Department of Pathology, Yale University School of Medicine, New Haven, CT 06510, USA.

## Abstract

**Images:**


British Journal of Cancer (1996) 74, 802-806
© 1996 Stockton Press All rights reserved 0007-0920/96 $12.00

### Functional assay for HER-2/*neu* demonstrates active signalling in a minority of HER-2/*neu*-overexpressing invasive human breast tumours

MP DiGiovanna[1,2], D Carter[1], SD Flynn[1] and DF Stern[1]

*Departments of [1]Pathology and [2]Internal Medicine, Section of Medical Oncology, Yale University School of Medicine and Yale-New Haven Hospital, 310 Cedar Street, New Haven, CT, 06510.*

**Summary** Overexpression of HER-2/*neu* in human breast carcinomas correlates with poor prognosis, although its strength as a prognostic indicator varies widely in different reports. Variability may be due to active signalling by HER-2/*neu* in a subset of the tumours in which it is overexpressed. To study this hypothesis, we have developed an activation state-specific anti-HER-2/*neu* monoclonal antibody. In this report, we use this antibody to analyse the signalling status of HER-2/*neu* in a large series of invasive breast carcinomas. Overexpression of HER-2/*neu* was detected in 9% of 223 cases. Of the cases demonstrating overexpression, active signalling by HER-2/*neu* was detected in only 35%. The clinicopathological characteristics of these cases are described. This functional assay is predicted to improve the utility of HER-2/*neu* as a prognostic indicator.

**Keywords:** HER-2/*neu*/c-*erb*B-2; receptor tyrosine kinase; breast cancer; oncogene; prognostic factor

HER-2/*neu* is overexpressed in 10–40% of human breast carcinomas, and is reported to correlate with adverse prognostic factors and to be an independent predictor of poor prognosis itself (reviewed in Hynes and Stern, 1994). However, the reported strength of such associations varies widely among different studies, and some find minimal prognostic ability of this marker. As a result, the utility of HER-2/*neu* as a prognostic indicator remains a matter of contention.

As level of expression is not absolutely indicative of functional status, one potential cause of conflicting results may be biological heterogeneity in the degree of signalling by HER-2/*neu* among individual HER-2/*neu*-overexpressing tumours. Like other receptor tyrosine kinases, signal transduction by HER-2/*neu* proceeds via receptor autophosphorylation, recruitment of other signalling molecules and substrate phosphorylation (reviewed in Hynes and Stern, 1994). Although signalling activity of overexpressed receptors increases in graded fashion as receptor abundance increases, the signalling activity even of overexpressed receptors is dramatically affected by ligand binding. Moreover, activation of receptors leads to down-regulation, resulting in a lower steady-state abundance. Thus, overall levels do not reflect the degree to which the receptor is signalling and by extension the extent to which it influences the behavior of the tumour.

To address this issue, we have developed a monoclonal anti-HER-2/*neu* antibody, designated PN2A, which has absolute specificity for the phosphorylated form of the receptor (DiGiovanna and Stern, 1995). As autophosphorylation is a hallmark of active signalling, PN2A is uniquely suitable for specifically detecting activated receptor. We have previously reported that among five cases of ductal carcinoma *in situ* (DCIS) with HER-2/*neu* overexpression, PN2A detected phosphorylated receptor in only two (DiGiovanna and Stern, 1995), in support of our hypothesis. In this work, we extend our observations by applying this functional assay to a large series of invasive breast tumours, and we describe the frequencies of such occurrences as well as their clinicopathological characteristics.

Correspondence: DF Stern, Yale University School of Medicine, Department of Pathology, BML 342, 310 Cedar Street, New Haven, CT06510 USA
Received 3 November 1995; revised 22 February 1996; accepted 7 March 1996

### Materials and methods

### Selection of cases

A total of 262 cases of invasive or mixed invasive and *in situ* breast carcinomas ascertained between 1992 and 1994 at Yale–New Haven Hospital (New Haven, CT, USA) were randomly selected from the archival tissue bank of the Department of Pathology. All blocks were paraffin sections that had been fixed in 10% neutral buffered formalin. The presence of invasive carcinoma was verified in haematoxylin and eosin (H&E) sections of each case by a surgical pathologist (DC). Nineteen blocks were found to have only *in situ* carcinoma (CIS) remaining, and another 20 blocks had no carcinoma at all remaining. These 39 cases were eliminated from data tabulation, leaving 223 cases for analysis.

### Immunohistochemistry

Immunohistochemical staining for HER-2/*neu* was performed essentially as described previously (DiGiovanna and Stern, 1995). The phosphorylation-insensitive anti-HER-2/*neu* antibody Ab3 (clone 3B5, Oncogene Science, Manhasset, NY, USA) was used exactly as described (DiGiovanna and Stern, 1995). Phosphorylation state-dependent anti-HER-2/*neu* monoclonal antibody PN2A was affinity purified and used at a concentration of 20 $\mu g\ ml^{-1}$. Antigen retrieval by the pressure cooking method (Norton *et al.*, 1994) was also used to enhance PN2A immunostaining. For both antibodies, only a membranous pattern of immunostaining was considered positive (see DiGiovanna and Stern, 1995 for a discussion of interpretation of staining patterns). The antigen retrieval method caused an increase in diffuse cytoplasmic staining with PN2A compared with staining in the absence of this step. We consider this cytoplasmic staining artefactual background, as it is generally not observed with the Ab3 staining and it is also seen in HER-2/*neu*-negative (i.e. Ab3-negative) tumours. It is not expected that phosphorylated receptor (PN2A staining) would be detectable in the absence of any detectable receptor expression (Ab3 staining). As expected, the first 20 Ab3-negative cases that were stained with PN2A were all found to be negative for PN2A membrane staining. For the remainder of the study, PN2A staining was performed only on Ab3-positive tumours. Each sample was scored semiquantitatively as to the intensity of the membranous staining on a four-point scale, with 0

**Figure 1** Immunohistochemical staining of breast carcinomas with anti-HER-2/*neu* antibodies Ab3 (left) and PN2A (right). Case 1, (**a** and **b**) Pure invasive ductal carcinoma positive for both Ab3 (**a**) and PN2A (**b**). Case 2, (**c** and **d**) Pure invasive ductal carcinoma positive for Ab3 (**c**) but negative for PN2A (**d**). [In (**d**) a diffuse light non-specific background was exaggerated by the filter used for black and white photography, although under direct microscopy the cellular details were clearer.] Case 3 (**e**–**h**) harbours invasive carcinoma positive for Ab3 (**e**) and PN2A (**f**), and CIS positive for Ab3 (**g**) and PN2A (**h**). Case 4 (**i**–**l**) harbours invasive carcinoma positive for Ab3 (**i**) but negative for PN2A (**j**), while it harbours CIS positive for both Ab3 (**k**) and PN2A (**l**). Original magnification × 100.

indicating absence of membrane staining, 1+ the least amount of staining detectable and 4+ representing the most intensely staining specimens. A tumour known to express phosphorylated HER-2/*neu* was run as a positive control with each batch.

Oestrogen receptor (ER) and progesterone receptor (PR) levels were assayed immunohistochemically at the time of surgical resection. The assay was performed by the Yale–New Haven Hospital clinical pathology laboratory using the respective antibodies and staining kits from Abbott Labs (North Chicago, IL, USA). Paraffin sections stained for ER were pretreated with pronase.

### *Flow cytometric analysis*

To determine the per cent S-phase and ploidy in formalin-fixed, paraffin-embedded tissue, the nuclear DNA content was analysed as described previously (Filderman *et al*., 1992).

### Results

A total of 223 randomly selected cases of invasive (or mixed invasive and CIS) human breast tumours were examined for overexpression of HER-2/*neu* by immunohistochemical staining of formalin-fixed, paraffin-embedded archival specimens. Membranous immunostaining with Ab3 indicative of HER-2/*neu* overexpression was detected in the invasive component in 20 cases, or 9% overall (Figure 1). The percentage of positively staining cases was roughly equivalent for all pathological categories, with the exception of negative staining for all (11) cases having lobular carcinoma as the only invasive component. For cases with an original diagnosis of invasive ductal carcinoma plus any CIS, both components were remaining in 74 of the blocks examined. Of these 74 blocks, the invasive and CIS components were concordant for HER-2/*neu* overexpression in all but six cases. There were three cases in which the CIS stained but the invasive component did not, and three cases in which the invasive component stained but the CIS did not.

Each of the 20 HER-2/*neu*-overexpressing cases was evaluated for activation of HER-2/*neu* by PN2A immunohistochemistry, and seven (35%) were found to be positive (Figure 1a and b compared with c and d). Two of the seven PN2A-positive cases also harboured HER-2/*neu*-overexpressing CIS, and the CIS was also PN2A positive (one case shown in Figure 1e–h). Of the 13 cases in which the invasive carcinoma was PN2A negative, three harboured HER-2/*neu*-overexpressing CIS, and two of these CIS components were PN2A positive (one case shown in Figure 1i–l). Although the numbers are small, these data suggest that when the CIS is not phosphorylated, the invasive component will also not be phosphorylated, but when the CIS is phosphorylated, the invasive component may or may not be. Thus, if there is a progression from CIS to invasive carcinoma, it appears that non-phosphorylated CIS remains non-phosphorylated upon invasion, but phosphorylated CIS gives rise to invasive carcinoma, which may remain phosphorylated or become unphosphorylated. Mechanistically, there may be increased phosphatase activity upon invasion or decreased ligand abundance in the microenvironment of the stroma. The inability to maintain activation upon invasion could remove the selective pressure for HER-2/*neu* overexpression, potentially explaining the well-described lower frequency of overexpression in invasive compared with pure *in situ* carcinoma. Most importantly, it is clear that HER-2/*neu* phosphorylation, a reflection of activation, occurs in only a subset of the invasive breast tumours that overexpress this receptor.

Consistent with other studies, overexpression of HER-2/*neu* showed a trend towards inverse association with ER (Table I), with a 45% rate of ER positivity among 'neu+' cases (9/20) *vs* a 59% rate of ER positivity among 'neu−' cases (120/203). Phosphorylated HER-2/*neu* (Pneu) was associated with a similar rate of ER positivity as was overall HER-2/*neu*. More strikingly, a statistically significant inverse association was found between HER-2/*neu* and PR (Table I), with a 20% rate of PR positivity among 'neu+' cases (4/20) *vs* a 52% rate of PR positivity among 'neu−' cases (105/201). In addition, in the case of PR, phosphorylated HER-2/*neu* demonstrated an even more dramatic inverse association, with PR present in 31% (4/13) of 'neu+/Pneu−' cases *vs* 0% (0/7) of 'neu+/Pneu+' cases. Thus, while HER-2/*neu* overexpression is inversely associated with hormone receptor

**Table I** Clinicopathological characteristics stratified by invasive component staining for overall HER-2/*neu*, phosphorylated HER-2/*neu* (Neu+/Pneu+) and non-phosphorylated HER-2/*neu* (Neu+/Pneu−).

| *Category* | *Neu− (%)* | *Neu+ (%)* | *Neu+/Pneu− (%)* | *Neu+/Pneu+ (%)* |
|---|---|---|---|---|
| ER+ | 120/203 (59) | 9/20 (45) | 6/13 (46) | 3/7 (43) |
| PR+ | 105/201 (52) | 4/20 (20)[a] | 4/13 (31) | 0/7 (0)[a] |
| LN+ | 49/143 (34) | 6/13 (46) | 4/9 (44) | 2/4 (50) |
| Nuclear grade | | | | |
| 1 | 5/174 (3)[b] | 0/19 (0) | 0/12 (0) | 0/7 (0) |
| 2 | 128/174 (74) | 10/19 (53) | 7/12 (58) | 3/7 (43) |
| 3 | 42/174 (24) | 9/19 (47) | 5/12 (42) | 4/7 (57) |
| Histological grade | | | | |
| 1 | 10/174 (6)[c] | 1/19 (5)[b] | 1/12 (8)[b] | 0/7 (0) |
| 2 | 85/174 (49) | 6/19 (32) | 4/12 (33) | 2/7 (29) |
| 3 | 81/174 (47) | 13/19 (68) | 8/12 (67) | 5/7 (71) |
| Ploidy | | | | |
| Diploid | 66/158 (42) | 3/10 (30) | 3/7 (43) | 0/3 (0) |
| Tetraploid | 27/158 (17) | 3/10 (30) | 2/7 (29) | 1/3 (33) |
| Aneuploid[d] | 65/158 (41) | 4/10 (40) | 2/7 (29) | 2/3 (67) |
| S-Phase | | | | |
| Low | 83/113 (73) | 4/6 (67) | 4/5 (80) | 0/1 |
| High | 30/113 (27) | 2/6 (33) | 1/5 (20) | 1/1 |
| Age (years) | | | | |
| <50 | 60/203 (30) | 8/20 (40) | 6/13 (46) | 2/7 (29) |
| ⩾50 | 143/203 (70) | 12/20 (60) | 7/13 (54) | 5/7 (71) |

[a]Statistically significant differences from 'Neu–' results by Chi-square test at $P<0.01$. [b]One case had two components of differing grades. [c]Two cases had two components of differing grades. [d]Other than tetraploid.

expression, it appears that activated, phosphorylated HER-2/*neu* correlates with absence of PR strikingly more strongly than does overall HER-2/*neu*.

As reported by others, we found that all 'neu+' cases showed trends towards higher nuclear grades, higher histological grades and lymph node positivity (Table I). Stratification by phosphorylation status using PN2A was notable for a trend towards higher nuclear grade when receptor is phosphorylated. HER-2/*neu*-overexpressing tumours also showed a tendency towards higher frequency of aneuploidy (Table I). For the three 'neu+/Pneu+' cases for which ploidy data were available, all three were aneuploid, whereas the 'neu+/Pneu−' cases showed a similar frequency of aneuploidy as the 'neu−' cases (Table I). No distinct trends were obvious for S-phase fraction analysis or age stratification.

One potential technical consideration of our studies is that PN2A staining may simply reflect the subpopulation of tumours with the highest levels of HER-2/*neu* overexpression. If all HER-2/*neu* were actually phosphorylated, but PN2A had significantly less sensitivity than Ab3 in detecting antigen under optimal conditions, then PN2A could simply identify the tumours with the highest levels of HER-2/*neu* overexpression. In that scenario, PN2A staining would be closely correlated with Ab3 staining. However, the overall level of receptor expression (Ab3 staining) by necessity defines the limits of detection of receptor phosphorylation (PN2A staining). It is also true that cell culture experiments have shown that the greater the level of HER-2/*neu* overexpression, the higher the basal level of receptor phosphorylation (Stern *et al.*, 1988). Therefore, even if the phosphorylation of HER-2/*neu* was completely random, a certain degree of association between Ab3 and PN2A would be necessary. We addressed this issue by examining semiquantitatively the intensity of PN2A staining for phosphorylated HER-2/*neu* in comparison with Ab3 staining for overall HER-2/*neu* on a four-point scale as described in Materials and methods. As shown in Figure 2, there is some variation between the two variables. Although there is a tendency for the strongest overexpressors to have detectable phosphorylation, consistent with cell culture experiments, there are examples of cases with low Ab3 HER-2/*neu* staining (1+ and 2+) that still have detectable PN2A staining, as well as cases with very strong HER-2/*neu* staining (3+ and 4+) that have undetectable PN2A staining. The Pearson's coefficient of correlation $r=0.339$, again demonstrating a weak correlation. Hence, PN2A positivity does not simply select for the highest overexpressing cases. Therefore, we conclude that PN2A detects HER-2/*neu* that has been specifically activated, either by agonistic ligand, transmodulating factors or by activating mutation (although the latter has not been reported in human breast carcinoma; Lemoine *et al.*, 1990).

### Discussion

In this report, we demonstrate that in invasive breast carcinoma HER-2/*neu* may exist either in a phosphorylated, and therefore actively signalling, state or in a non-phosphorylated and presumably inactive state. Tumours expressing activated HER-2/*neu* are strikingly PR negative, as well as possibly having a tendency towards higher nuclear grade and aneuploidy, although a larger series of HER-2/*neu*-overexpressing tumours will be required to verify these findings. These results confirm and refine additional previous results from our laboratory employing image analysis using antiphosphoreceptor polyclonal antibody A1, which recognizes both phosphorylated HER-2/*neu* and epidermal growth

**Figure 2** Scatter plot of intensity of immunohistochemical staining with PN2A as compared with Ab3. Pearson's coefficient of correlation $r=0.339$.

factor receptor (Bacus *et al.*, 1996). In that study, which was performed on frozen sections non-randomly selected such that most overexpressed HER-2/*neu*, we also found a moderate correlation between staining for HER-2/*neu* and phosphorylated receptors, and we found HER-2/*neu* and phosphorylated receptor scores to correlate inversely with PR.

In the present study, the overall frequency of HER-2/*neu* overexpression (9%) is in the lower range of what has generally been reported in the literature for invasive carcinomas (often up to 20%). One potential reason is that the Ab3 antibody may have a lower sensitivity than antibodies used in other studies. In a comparison of sensitivities of a large panel of antibodies to HER-2/*neu* by Press *et al.* (1994), Ab3 (clone 3B5) had 61% the sensitivity of the most sensitive antibody and an estimated 50% sensitivity compared with the 'ideal result' (it also had a 98% specificity). We chose this antibody because it was prepared against a peptide of the same amino acid sequence as PN2A. Thus, staining with Ab3 assured that the PN2A epitope was intact.

In summary, we have demonstrated that HER-2/*neu* is phosphorylated, and therefore actively signalling, in a minority of the invasive breast carcinomas overexpressing this receptor. As a non-functioning receptor is unlikely to influence the phenotype of a tumour regardless of its level of expression, we predict that tumours harbouring activated HER-2/*neu* probably have a significantly more aggressive clinical course, and that those harbouring inactive receptors probably do not differ from those lacking receptor overexpression. A retrospective analysis of a large series of breast tumours with long-term follow-up using this novel functional assay is under way in our laboratory to test this prediction.

**Acknowledgements**
The authors thank Tracey Davison and Dr Chris Howe of the Yale University Critical Technologies Program for procurement of human breast tumour samples. Melissa A Lerman assisted with immunohistochemical staining. This work was supported in part by grants from the Mathers Foundation and USPHS RO1-CA45708. MPD has a postdoctoral fellowship research award from the United States Army Medical Research and Development Command, Grant DAMD17-94-J-4135. The content of the information contained herein does not necessarily reflect the position or the policy of the United States Government, and no official endorsement should be inferred.

### References

BACUS SS, CHIN D, YARDEN Y, ZELNICK CR AND STERN DF. (1996). Type 1 receptor tyrosine kinases are differentially phosphorylated in mammary carcinoma and differentially associated with steroid receptors. *Am. J. Pathol.*, **148**, 549–558.

DIGIOVANNA MP AND STERN DF. (1995). Activation state-specific monoclonal antibody detects tyrosine phosphorylated p185$^{neu/erbB\text{-}2}$ in a subset of human breast tumors overexpressing this receptor. *Cancer Res.*, **55**, 1946–1955.

FILDERMAN AE, SILVESTRI GA, GATSONIS C, LUTHRINGER DJ, HONIG J AND FLYNN SD. (1992). Prognostic significance of tumor proliferative fraction and DNA content in stage I non-small cell lung cancer. *Am. Rev. Respir. Dis.*, **146**, 707–710.

HYNES NE AND STERN DF. (1994). The biology of *erb*B-2/*neu*/*HER*-2 and its role in cancer. *Biochim. Biophys. Acta*, **1198**, 165–184.

LEMOINE NR, STADDON S, DICKSON C, BARNES DM AND GULLICK WJ. (1990). Absence of activating transmembrane mutations in the *c-erbB*-2 proto-oncogene in human breast cancer. *Oncogene*, **5**, 237–239.

NORTON AJ, JORDAN S AND YEOMANS P. (1994). Brief, high-temperature heat denaturation (pressure cooking): a simple and effective method of antigen retrieval for routinely processed tissues. *J. Pathol.*, **173**, 371–379.

PRESS MF, HUNG G, GODOLPHIN W AND SLAMON DJ. (1994). Sensitivity of HER-2/*neu* antibodies in archival tissue samples: Potential source of error in immunohistochemical studies of oncogene expression. *Cancer Res.*, **54**, 2771–2777.

STERN DF, KAMPS MP AND CAO H. (1988). Oncogenic activation of p185$^{neu}$ stimulates tyrosine phosphorylation in vivo. *Mol. Cell. Biol.*, **8**, 3969–3973.